# Research in advance for FMD Novel Vaccines

**DOI:** 10.1186/1743-422X-8-268

**Published:** 2011-06-03

**Authors:** Liang Zhang, Jie Zhang, Hao-tai Chen, Jian-hua Zhou, Li-na ma, Yao-zhong Ding, Yong-sheng Liu

**Affiliations:** 1 State Key Laboratory of Veterinary Etiological Biology, National Foot-and-Mouth Disease Reference Laboratory, Lanzhou Veterinary Research Institute, Chinese Academy of Agricultural Sciences, Lanzhou, Gansu, China

## Abstract

Foot-and-Mouth Disease (FMD), as a major global animal disease, affects millions of animals worldwide and remains the main sanitary barrier to the international and national trade of animals and animal products. Inactivated vaccination is the most effective measure for prevention of FMD at present, but fail to induce long-term protection and content new requires for production of FMD vaccines. As a number of Researchers hope to obtain satisfactory novel vaccines by new bio-technology, novel vaccines have been studied for more than thirty years. Here reviews the latest research progress of new vaccines, summarizes some importance and raises several suggestions for the future of FMD vaccine.

## 1 Introduction

Foot-and-mouth disease (FMD) is a severe, highly contagious and economically devastating viral disease worldwide (Table 1), which affects animals with cloven hoofed animals, such as cattle, pigs, deer, goats and sheep. It has been reported many outbreaks around the world since FMD firstly broke out in America in 1870. Although not usually fatal, FMD, as a renewed public and political high-profile disease, has aroused the global concerns [1,2]. It not only reduces animals' commercial value by decreasing animals' weight and milk output, but is also the most important animal disease limiting commerce of animals and animal products [3]. For those reasons, it is significant to prevent, control and even eradicate FMD.

**Table 1:** Various serotypes in FMDV distribution areas

Area	Main serotypes in FMDV distribution areas						
	Type O	Type A	Type C	Type Asia I	SAT-1	SAT-2	SAT-3
China	+	+	-	+	-	-	-
Southeast Asia	+	+	-	+	-	-	-
Africa	+	-	-	-	+	+	+
Middle East	+	+	+	+	-	-	-
United Kingdom	+	-	-	-	-	-	-
South America	+	+	+	-	-	-	-

"+" means positive, and "-" means negative.

Vaccines, available since the early 1900s, have been the most instrumental method for prevention and control of FMD. Nowadays, FMD vaccine is produced by growing live velogenic foot-and-mouth disease virus (FMDV) in BHK-21 cell cultures under bio-secure conditions and inactivating it by using a chemical such as binary ethyleneimine. However, the mode of production exist a risk to reveal live FMDV to environment. The risk should be considered by government when FMD has been effectively controlled. Moreover, at the beginning of the 21st century, the protocol for production of inactivated FMD vaccines allows the use of serological tests that can differentiate infected from vaccinated animals, formulation of vaccines that include multiple serotypes and subtypes and a number of adjuvants [4]. Besides, there are other important shortcomings of current inactivated vaccines, including short shelf life, the need for adequate cold chain of formulated vaccines, and difficulties of certain serotypes and subtypes to grow well in cell culture for vaccine production [5]. In addition, a risk from epidemic areas still exists in countries and areas having been free of FMDV. In order to address these problems, new vaccines are necessary and urgent to develop into satisfied candidates of classical vaccines. According to mechanism, form and source of vaccine, this article reviewed the latest research progress of novel vaccines and some viewpoints for the development of FMD vaccines.

## 2 subunit vaccine

Subunit vaccine is a vaccine containing viral antigens made free of viral nucleic acid by chemical extraction or bio-expressing and containing only minimal amounts of non-viral antigens derived from the culture medium. It is less likely to cause adverse reactions than a vaccine containing the whole virion.

With the development of information concerning viral capsid structure, researchers had determined that VP1, one of the FMDV capsid proteins, had a prominent surface exposure in 1970s [6,7]. Based on this information and related researches, a number of strategies were designed to develop subunit vaccines as alternatives to conventional inactivated vaccine. In 1975, Bachrach et al. firstly obtained VP1 isolated from purified viruses inducing a neutralizing antibody response in swine [8]. In 1981, Kupper cloned the VP1 gene, transferred it into E.coli in the form of recombinant plasmid and harvested the VP1 [9]. Results indicated that VP1 produced in E. coli could protect both swine and cattle from virus challenge [10]. Wang JH, et al. refolded the VP1 with the assistant of SDS and obtained purified VP1 and induced neutralizing antibody responses to every refolding VP1. There was a protective immune response against FMDV challenge in guinea pigs vaccinated with recombinant P1 polyprotein expressed in Pichia pastoris [11]. Shi et al. verified fusion protein of bovine IFN-γand FMDV VP1 antigen expressed in P. pastoris could induce an immune response to FMDV antigens [12].

To study FMDV structural proteins and non-structure proteins (NSP), researchers screened several epitopes of VP1. Luis et al. found that TrpE fusion proteins containing portions of the C -terminal region of FMDV VP1 can induce a neutralizing antibody response and certain protection in animal test [13]. Subsequently, Yang and Zhuang separately tested the immune effect of fusion proteins including T cell and B cell epitopes. The results indicated that fusion proteins could cause humoral and cellular immune response [14,15].

Empty viral capsids are virus particles lacking nucleic acid which are naturally produced in infected cells and are as immunogenic as virions [7]. In 1979, Rweyemamu confirmed stability and immunogenicity of empty particles of FMDV. Subsequent studies reviewed in vitro expresstion of P1-2A and 3C in cells reformed 76s virus-like particles [16]. And Li et al. screened MDBK cells stably co-expressing the capsid precursor protein P1-2A gene and the protease 3C gene of FMDV [17]. The immune effective of virus-like particles is similar to whole FMDV.

Recently new subunit vaccines for FMDV were frequently reported, however, there were no subunit vaccines revealing advanced effective compared with traditional inactivated vaccines. In spite of it, subunit vaccines are still widely regarded as optimal candidates instead of classical inactivated vaccines for advantages, such as high security and the use of serological tests that can differentiate infected from vaccinated animals [4,18]. This ideal can be most likely achieved by the development of empty capsid vaccines in future. Utilizing the baculovirus-silkworm expression system, Li et al. developed recombinant virus vaccine, the immunological assays of guinea pigs demonstrated that this vaccine showed good immunogenicity and long immune persistence.

During the analysis and test of them, some subunit vaccines induced limited protection against virus. According to the characterization of various target protein, it is necessary for subunit vaccines to select an appropriate expression system, such as Bacillus subtilis [19], Cyanobacteria [20], Filamentous fungus [21], Escherichia coli [9], Pichia pastoris [22], Baculovirus [23], Mammalian cell and so on. The last four expression systems of them have been utilized to the development of FMD vaccines. As FMDV is further detailed and expression systems are optimized and improved, the development of subunit vaccines will be promoted.

## 3 live vector vaccine

A live vector vaccine is a vaccine that uses a chemically weakened virus to transport pieces of the virus to stimulate an immune response. Adenovirus [24] and poxvirus are widely utilized to efficiently express heterologous genes.

The structure and function of Adenovirus genome has been studied thoroughly. Searchers exploited replication-defective adenovirus serotype 5 (Ad5) as live vector carrying 5 kb~8 kb DNA fragment. Juilliard et al. verified that a single immunization with a replication-defective adenovirus recombinant vector induces long-lasting humoral and cellular immune responses specific to the transgenic product [25,26]. Sanz-Parra cloned FMDV P1 gene into Ad5 genome. The animal test displayed that recombinant viruses induced cellular but not humoral antiviral immunity and partial protection in pigs [27]. Mayr developed replication-defective Ad5 containing the capsid and 3C protease coding regions of foot-and-mouth disease virus as a vaccine candidate [28]. The replication-defective Ad5, encoding the FMDV capsid coding region and an inactive form of the 3C proteinase, induced generated high levels of FMDV-neutralizing antibodies 4 weeks later resulting in complete protection of five of the six swine and limited disease in the remaining animal [28]. Early protection against homologous challenge was provided to swine inoculated replication-defective Ad5 expressing capsid proteins of FMDV strain A24 [29]. Correlational researches adenovirus-vectored FMDV vaccine also aroused a rapid protection of cattle from direct challenge with FMDV [30]. Recently studies indicated that there was effective protection of guinea pigs and swine by a recombinant adenovirus expressing O serotype of foot-and-mouth disease virus whole capsid and 3C protease [31].

Some researchers had an attempt to exploit recombinant vaccinia virus [32] fowlpox virus [33] and pseudorabies virus [34] expressing FMDV structural protein, non-structure protein or both of them, but swine and guinea pigs inoculated with multiple doses were partially protected because of their low level of expression. However, FMD vaccines are required to protect animals from FMDV challenge by a single dose of vaccine in the epidemic period. Although the live vector vaccines appear promising, they are still at earlier stages of testing and development compared to the subunit vaccine candidates.

## 4 Nucleic acid vaccines

Nucleic acid vaccines are genetically engineered DNA to produce an immunological response in injected animals. It has been applied to a number of viral, bacterial and parasitic disease models as well as several tumour models. There are a number of advantages on nucleic acid vaccines over conventional vaccines, such as long-term protection, long shelf life [35] and the ability to induce a wider range of immune response types.

Plasmids have been used for development of DNA vaccines as ideal materials. Plasmids encoding FMDV structural protein and non-structural protein elicited immune responses in mice and swine and protected swine against viral infection [36]. Same other researches reviewed same results [37]. One of the hotspots of new studies of DNA vaccine is focus on the influence of cytokine. Multiple cytokine has been tested as adjuvants of DNA vaccines. There is Table 2 showing the influence of various cytokine on FMDV DNA vaccine [12,38-44]. Based on a large number of tests, some cytokines were identified as effective adjuvants of DNA vaccines. In further researches and development of DNA vaccines, cytokines will play a key role.

**Table 2:** The influence of various cytokine on FMDV DNA vaccine

Cytokine	Effective on FMDV DNA vaccine
IL-1/IL-2	Advanced the humoral immune response induced by FMD inactivated vaccines. [38,39]
IL-6	Advanced the cellular immune response induced by FMD DNA vaccines, Promoting the maturation and immune function of dendritic cells [40]
IL-9	Increased a robust antigen specific cytotoxicity T lymphocyte response [41]
IL-15	Enhanced the cellular and mucosal immune response and the level of IFN-γinduced by FMD DNA vaccines [42]
IL-18	Increased the immunogenicity of DNA vaccines coding P12A and 3C of FMDV [43]
CSF	Enhanced immune responses against a plasmid DNA vaccine encoding FMDV empty capid [44]
INF-α/β	Advanced the cellular immune response induced by FMD DNA vaccines, Promoted the maturation and immune function of dendritic cells
INF-γ	Enhanced both humoral and cell-mediated immune responses[12]

## 5 Novel attenuated vaccine

Novel attenuated vaccines are engineered to knock out some regions or oligonucleotides of viruses by biotechnology but not continuously cultured in a non-native susceptible host just as traditional attenuated vaccines. Compared with classical attenuated vaccines, new attenuated methods are stable and low risk of toxicity reversion. There are mainly two strategies exploited to develop novel attenuated vaccines. Based on the researches of FMDV receptor, receptor binding site-deleted (or replaced) FMDV attenuated vaccine has been explored to protect cattle from FMD [45,46]. On the basis of experimental researches into non-required for viral replication, live-attenuated vaccines prepared from a leader proteinase-deficient serotype A12 FMDV [47-49] provided an effective protection to cattle from the challenge of FMDV. These series of experiments demonstrate the potential of a rationally designed live-attenuated FMDV vaccine.

With deeply fundamental researches on the structure and functionality of FMDV proteins, rising mechanisms of virulence and pathogenicity have been reviewed. The leader proteinase of FMDV inhibits the induction of beta interferon mRNA and blocks the host innate immune response [50]. But little is known about other virulence determinants among FMDV genome. As new virulence determinants are identified, the artificial attenuated vaccines will have a further development on condition that this evaluation system of the possibility of reversion to virulence is established.

## 6 Synthetic peptide vaccine

Synthetic peptide vaccine is a viral peptide synthesized by chemical approaches, including antigen epitopes. With the development of information concerning FMDV antigen determinants, antigen regions represented the variable G-H loop found on the surface of the FMDV capsid [51,52] and the carboxy-terminal region of VP1 and corresponded to B cell epitopes. In an earlier experiments [53-55], peptide vaccines against a single epitope of FMD just indicated the limited immunogenicity and particle protection. Further researches implied that an effectively protective VP1 peptide vaccine needs the addition of promiscuous Th sites from a source outside VP1 [56-58] and potent B cell sites for the induction of high affinity neutralising antibodies [59,60]. On the basis of the above researches, researchers have designed novel synthetic peptide vaccines with T and B cell sites optimised for both immunogenicity and antigenic cross-reactivities. This peptide immunogen spans the entire G-H loop domain and extensive flanking sequences (129-169), has a unique consensus sequence to confront the hypervariability of serotype O viruses, and includes a promiscuous artificial Th site [61]. A dendrimeric peptide vaccine was described to protect pigs against challenge with FMDV and induce high titers of FMDV neutralizing antibodies, activated FMDV-specific T cells and a potent anti-FMDV immunoglobulin A response [62]. The research suggested that peptide vaccines representing appropriate advanced structure preferably more possibly induced a fully protective immune response, and rational designs of advanced structure of antigen peptide would provide a new development of peptide vaccines.

## Conclusion

The globalization of commerce is accelerating the spread of FMDV and presents new requirements on the trade of animals and animal productions. But novel vaccines against FMDV are developed slowly, and only few available novel FMD vaccines have been used in practice. New vaccines are still just tested and evaluated in laboratory and clinic, due to limited details on immune mechanism of each of novel vaccines and the basic researches of FMDV. However, a number of results implied that there were observed advantages and disadvantages on novel vaccines arrange the security of production, the security of vaccinated animal, shelf life, duration of immune response, vaccination effectiveness and differentiation of infected animals from vaccinated ones(Table 3).

**Table 3:** Advantages and disadvantages of different novel vaccines

Novel vaccines	security of production	security of vaccinated animal	shelf life	duration of immune response	vaccination effectiveness	Differentiation of infected animals from vaccinated ones
Subunit vaccine	Yes	Yes	Normal	Normal	Low	Yes
Live vector vaccine	Yes	Yes	Normal	Long	High	Yes
Nucleic acid vaccine	Yes	Risk to recombinant to other genomes	Long	Long	Low	Yes
Novel attenuated vaccine	Yes	Risk to toxicity reversion but low	Normal	Long	High	Yes
Synthetic peptide vaccine	Yes	Yes	Normal	Short	Low	Yes

We should also do some efforts to the development of adjuvants and immunization strategies. Some researchers reported that combination immunization with DNA vaccines and subunit vaccines or synthetic peptide vaccines could induced a high-titer specific antibodies and cellular immunity. This strategy will be a nearest way to make different vaccines complement each other and proceed to the practice of novel vaccines, on the condition that researchers are puzzled to develop a single novel vaccine for controlling FMDV.

## Competing interests

The authors declare that they have no competing interests.

## Authors' contributions

LZ and JZ contributed equally to the original draft of the manuscript, and approved the final version. HTC and JHZ helped to provide information and suggestion. LNM and YZD contributed to conception and design of the manuscript, and revised the manuscript. YSL is the corresponding author. All authors read and approved the final manuscript.
